# Retrograde Cerebral Perfusion in Type A Aortic Dissection Repair Is Associated With Higher Rates of Postoperative Complications

**DOI:** 10.7759/cureus.115458

**Published:** 2026-08-30

**Authors:** Abhishek Chakraborty, Kevin Hayes, Hirohisa Ikegami, Gengo Sunagawa, Mark J Russo, Leonard Y Lee, Anthony Lemaire

**Affiliations:** 1 Cardiac Surgery, Rutgers Robert Wood Johnson Medical School, New Brunswick, USA

**Keywords:** antegrade cerebral perfusion, cerebral protection, deep hypothermic circulatory arrest, retrograde cerebral perfusion, type a aortic dissection

## Abstract

Background

The comparative short- and long-term outcomes of retrograde versus antegrade cerebral perfusion during circulatory arrest for acute type A aortic dissection repair remain debated.

Methods

In this single-center retrospective study, postoperative outcomes were compared between retrograde and antegrade cerebral perfusion. Retrograde cerebral perfusion was employed in older patients (median age = 72 vs. 63 years; p = 0.030), with comparable body mass index (29 vs. 28 kg/m²; p = 0.649).

Results

Relative to antegrade cerebral perfusion, retrograde cerebral perfusion was associated with increased 30-day mortality (40% vs. 13%; p = 0.003), postoperative pericardial and pleural effusion (75% vs. 43%; p = 0.009), and cerebrovascular accident (35% vs. 16%; p = 0.044). After multivariable adjustment for circulatory arrest time and nadir hypothermic temperature, retrograde perfusion independently predicted 30-day mortality (adjusted odds ratio: 7.66; 95% CI: 2.16-27.13; p = 0.002).

Conclusions

These findings suggest that retrograde cerebral perfusion is associated with worse postoperative outcomes following acute type A aortic dissection repair and warrant further investigation.

## Introduction

Acute type A aortic dissection (ATAAD) is a life-threatening surgical emergency characterized by blood entering an intimal tear, resulting in the separation of the intima from the media and creation of a false lumen. This pathologic cascade usually precipitates profound hemodynamic instability, end-organ malperfusion, and a markedly increased risk for aortic rupture [1,2]. Operative repair often necessitates deep hypothermic circulatory arrest (DHCA), which decreases the metabolic demand of ischemia-sensitive tissues, such as the brain, during the period of total-body circulatory arrest [3,4]. Antegrade cerebral perfusion (ACP), achieved through selective cannulation of the axillary or innominate artery, has become an increasingly prevalent adjunct during circulatory arrest for the purpose of neuroprotection. Similarly, retrograde cerebral perfusion (RCP) accomplished through direct cannulation of the superior vena cava is an alternative option for neuroprotection.

Despite the widespread use of both techniques, the optimal cerebral perfusion strategy remains debated, with selection often guided by institutional preference rather than definitive evidence [5-7]. Recent retrospective studies have shown no difference in outcomes, including 30-day mortality, hospital length of stay, and neurological complications. Likewise, the only randomized clinical trial comparing RCP and ACP demonstrated no difference in all-cause neurologic mortality, stroke, or cognitive outcomes following ATAAD [8-10]. Even with this evidence, the adoption of ACP for ATAAD repair is steadily increasing [7].

Much of the existing evidence, including the aforementioned studies, derives from heterogeneous cohorts that combined elective with emergent presentations and pooled data across various institutions and surgical eras, which may potentially obfuscate technique-specific outcomes. A uniform single-center cohort, in which perioperative management, surgical technique, and temperature strategy are consistently similar, may therefore be better positioned to isolate differences attributable to perfusion approach. As survival following ATAAD repair improves, long-term outcomes assume greater importance. Thus, evaluation of cerebral perfusion strategy is warranted, particularly in the absence of robust clinical guidelines [11,12].

The objective of this study was to directly compare early postoperative outcomes between ACP and RCP in a uniform, single-center cohort of patients undergoing emergent ATAAD repair. By restricting the analysis to a uniform, single-center, emergent-only cohort, this study limits the influence of perfusion strategy from the case mix and inter-institutional heterogeneity that limit prior multi-institutional pooled analyses.

## Materials and methods

A retrospective review was performed of patients undergoing emergent repair of ATAAD between January 2016 and March 2026 at a single academic institution serving as a tertiary referral center for cardiovascular disease in New Jersey.

All consecutive patients undergoing repair of type A aortic dissection who received adjunctive selective cerebral perfusion during hypothermic circulatory arrest were included. The decision to pursue ACP vs. RCP was based on individual surgeon discretion. No standardized or prespecified criteria governed the decision. Cerebral perfusion was established either by placement of a balloon occlusion catheter directly into the innominate artery for antegrade perfusion or by cannulation of the superior vena cava for retrograde perfusion, targeting a perfusion pressure of approximately 50 to 60 mmHg for antegrade and 20 to 30 mmHg for retrograde perfusion. Systemic cooling was performed to a nadir temperature of approximately 25°C prior to circulatory arrest. During DHCA, the head was packed externally in ice and corticosteroids were administered at standard dosing in all patients, and cerebral oxygenation was monitored using cerebral oximetry. Following circulatory arrest, patients were rewarmed while maintaining a temperature gradient of no more than 10°C between the perfusate and the patient until a bladder temperature of 36°C was reached, with bladder temperature serving as the reference for rewarming. Adult patients (≥18 years) undergoing emergent ATAAD repair with hypothermic circulatory arrest and adjunctive ACP or RCP were eligible for inclusion. Patients who underwent elective aortic aneurysm repair, did not receive adjunctive cerebral perfusion, or had incomplete primary-outcome data were excluded. The sample size was determined by the number of consecutive patients who met inclusion criteria during the study period. All eligible patients were included. Patients were stratified into ACP and RCP cohorts according to the cerebral protection strategy employed during deep hypothermic circulatory arrest. The primary outcomes were 30-day (or in-hospital) mortality, postoperative length of stay, and 30-day readmission. Secondary outcomes were postoperative cerebrovascular accident, defined as a new clinically evident neurological deficit confirmed on imaging, acute kidney injury, postoperative pericardial and/or pleural effusion, and new-onset postoperative atrial fibrillation. All study outcomes were ascertained by structured review of the electronic medical record. Categorical variables are presented as counts (percentages) and were compared using the Pearson chi-squared test, or the Fisher exact test where expected cell counts were small; continuous variables are presented as medians (interquartile ranges) and were compared using the Mann-Whitney U test. Potential confounders, including patient age, circulatory arrest time, and nadir hypothermic temperature, were identified. A multivariable logistic regression model adjusting for circulatory arrest time and nadir hypothermic temperature was performed to control for the effects of these operative characteristics on outcomes. All tests were two-sided, and statistical significance was defined as p < 0.05. Analyses were performed using JMP (JMP Statistical Discovery LLC, Cary, NC) and Python (Python Software Foundation, Wilmington, DE).

## Results

A total of 116 patients underwent emergent ATAAD hemiarch repair during the study period, of whom 20 (17%) received RCP and 96 (83%) received ACP. Patients receiving retrograde perfusion were significantly older than those receiving antegrade perfusion (median age = 72 vs. 63 years; U = 656, p = 0.030), whereas body mass index was comparable between groups (29 vs. 28 kg/m²; U = 729, p = 0.649) (Table 1). No significant differences were observed in sex distribution (60% vs. 74% male; n = 12 vs. n = 71; χ²(1) = 1.58, p = 0.208) or in the prevalence of comorbidities. Nadir hypothermic temperature differed modestly between cohorts (median = 25°C in both; U = 1161, p = 0.027), while cardiopulmonary bypass, circulatory arrest, and cross-clamp times were comparable (Table 1).

**Table 1:** Baseline and perioperative characteristics of patients receiving retrograde versus antegrade cerebral perfusion. Continuous variables are reported as median (interquartile range) and were compared using the Mann–Whitney U test; categorical variables are reported as n (%) and were compared using the Pearson chi-squared test, or the Fisher exact test where expected cell counts were <5. * P < 0.05. ACP, antegrade cerebral perfusion; RCP, retrograde cerebral perfusion; IQR, interquartile range; COPD, chronic obstructive pulmonary disease.

Variable		Overall (n = 116)	Retrograde (n = 20)	Antegrade (n = 96)	P-value
Baseline characteristics					
	Age (years) (Median, IQR)	64 (54 – 73)	72 (57 – 77)	63 (54 – 69)	.030*
	Gender (male), n (%)	83 (72%)	12 (60%)	71 (74%)	.208
	Body mass index (Median, IQR)	28 (25 – 32)	29 (25 – 36)	28 (25 – 31)	.649
Comorbidities					
	Hypertension, n (%)	90 (78%)	14 (70%)	76 (79%)	.371
	Hyperlipidemia, n (%)	44 (38%)	8 (42%)	36 (38%)	.706
	Diabetes mellitus type I/II, n (%)	15 (13%)	2 (10%)	13 (14%)	.668
	Preoperative atrial fibrillation, n (%)	29 (25%)	7 (35%)	22 (23%)	.268
	Previous cerebrovascular accident, n (%)	11 (10%)	2 (11%)	9 (10%)	.898
	History of congestive heart failure, n (%)	11 (10%)	2 (10%)	9 (9%)	.942
	History of smoking, n (%)	35 (30%)	4 (20%)	31 (33%)	.264
	Diagnosis of COPD, n (%)	9 (8%)	1 (5%)	8 (8%)	.612
	History of coronary artery disease, n (%)	26 (32%)	5 (33%)	21 (31%)	.881
Perioperative characteristics					
	Cardiopulmonary bypass time (minutes)	143 (120 – 191)	152 (133 – 188)	139 (116 – 190)	.399
	Circulatory arrest time (minutes) (Median, IQR)	22 (18 – 27)	22 (19 – 26)	22 (18 – 28)	.515
	Cross-clamp time (minutes) (Median, IQR)	91 (72 – 124)	102 (58 – 120)	90 (74 – 125)	.509
	Hypothermia temperature (Median, IQR)	25 (24 – 26)	25 (24 – 25)	25 (24 – 26)	.027*

Postoperative outcomes are summarized in Table 2. Relative to the antegrade cohort, the retrograde cohort demonstrated significantly higher 30-day mortality (40% vs. 13%, n = 8 vs. n = 12; χ²(1) = 8.61, p = 0.003), postoperative pericardial and/or pleural effusion (75% vs. 43%, n = 15 vs. n = 41; χ²(1) = 6.91, p = 0.009), and cerebrovascular accident (35% vs. 16%, n = 7 vs. n = 15; χ²(1) = 4.04, p = 0.044). Postoperative length of stay and 30-day readmission did not differ significantly between cohorts. No significant differences were observed in acute kidney injury (20% vs. 29%, n = 4 vs. n = 28; χ²(1) = 0.70, p = 0.404) or postoperative atrial fibrillation (20% vs. 41%, n = 4 vs. n = 39; χ²(1) = 3.02, p = 0.082). After multivariable adjustment for circulatory arrest time and nadir hypothermic temperature, retrograde perfusion remained an independent predictor of 30-day mortality (adjusted odds ratio = 7.66; 95% CI = 2.16-27.13; p = 0.002).

**Table 2:** Postoperative outcomes and complications of patients receiving retrograde versus antegrade cerebral perfusion. Categorical variables are reported as n (%) and were compared using the Pearson chi-squared test or the Fisher exact test where expected cell counts were <5. Length of stay is reported as median (interquartile range) and was compared using the Mann-Whitney U test. * P < 0.05. ACP, antegrade cerebral perfusion; RCP, retrograde cerebral perfusion; IQR, interquartile range.

Variable		Overall (n = 116)	Retrograde (n = 20)	Antegrade (n = 96)	P-value
Outcomes					
	30-day mortality, n (%)	20 (17%)	8 (40%)	12 (13%)	.003*
	Postoperative length of stay (days) (Median, IQR)	8 (5 – 15)	8 (4 – 12)	8 (5 – 15)	.866
	30-day readmission, n (%)	23 (20%)	1 (5%)	22 (23%)	.079
Postoperative complications					
	Acute kidney injury, n (%)	32 (28%)	4 (20%)	28 (29%)	.404
	Postoperative pericardial/pleural effusion, n (%)	56 (48%)	15 (75%)	41 (43%)	.009*
	Postoperative cerebrovascular accident, n (%)	22 (19%)	7 (35%)	15 (16%)	.044*
	Postoperative atrial fibrillation, n (%)	43 (37%)	4 (20%)	39 (41%)	.082

## Discussion

This single-center analysis compared postoperative outcomes among patients undergoing ATAAD repair utilizing deep hypothermic circulatory arrest with either ACP or RCP. RCP was associated with significantly higher 30-day mortality, cerebrovascular accident, and pericardial/pleural effusion relative to ACP.

The RCP cohort was significantly older than the ACP cohort. Although advanced age has traditionally been regarded as a risk factor for in-hospital mortality following ATAAD repair, a systematic review by Harky and colleagues observed that age is becoming a progressively weaker predictor as surgical technique and perioperative care advance, with comorbidities such as hypertension and diabetes assuming a more prominent role in postoperative morbidity and mortality [13,14]. Notably, the excess 30-day mortality in the RCP cohort persisted after multivariable adjustment for circulatory arrest time and nadir hypothermic temperature (adjusted odds ratio: 7.66; 95% CI: 2.16-27.13; p = 0.002), and the two groups were otherwise comparable in body mass index and comorbidity burden. This pattern suggests that the mortality difference is not primarily attributable to baseline demographics but rather to the perfusion strategy itself.

Recent investigations have examined the influence of intraoperative cerebral malperfusion on survival [15,16]. Using ultrasonography of the internal carotid arteries, Furukawa and colleagues identified isolated cerebral malperfusion in 16 of 137 patients (11.7%) undergoing ATAAD repair despite physiologic flow delivered via antegrade perfusion [17]. Given that retrograde perfusion delivers non-physiologic flow, and that multiple large-animal models have demonstrated its inability to adequately perfuse the cerebral parenchyma [18,19], retrograde perfusion may predispose tissues to malperfusion-like injury, consequently elevating mortality relative to antegrade perfusion. Although recent human studies have not detected perfusion differences between the techniques, further investigation is required to characterize these deficits.

ACP restores physiologic flow through the cerebral vasculature but is not as adept at clearing atherosclerotic debris or air emboli and also necessitates manipulation of the arch vessels for cannulation. Retrograde perfusion, by contrast, has been proposed to flush metabolites, atheromatous debris, and emboli from the cerebral arterial vasculature, albeit at the cost of inferior tissue perfusion owing to venous shunting [5,20-23]. Notwithstanding prior large-animal and clinical evidence suggesting that retrograde perfusion confers protection against embolic neurologic injury relative to circulatory arrest alone and to antegrade perfusion, the present analysis paradoxically identified a higher incidence of postoperative cerebrovascular accident in the RCP cohort [20-23]. A plausible explanation is that disturbed or reversed flow, as occurs during retrograde perfusion, provokes endothelial injury that promotes thrombus formation, with subsequent dislodgement or vessel occlusion upon restoration of physiologic flow. Stasis within non-perfused capillaries may likewise precipitate microthrombosis and cerebral infarction.

Finally, the RCP cohort exhibited higher rates of postoperative pericardial and pleural effusion. Although postoperative pericardial effusion is recognized to occur more frequently after aortic procedures than after other cardiac operations, the present findings uniquely implicate the choice of cerebral perfusion strategy in effusion development [24,25]. During antegrade perfusion, oxygenated blood is delivered through the innominate or axillary artery at a perfusion pressure of usually 50 to 60 mmHg. Retrograde perfusion, conversely, requires oxygenated blood to be delivered to cerebral vessels through the superior vena cava, usually at 20 to 30 mmHg, to sustain adequate cerebral perfusion pressure [26]. Because the perfusion pressure required to maintain adequate cerebral flow exceeds physiologic right atrial pressure, secondary tricuspid regurgitation may develop, flowing into the pulmonary arteries. The subsequent elevation in pulmonary capillary hydrostatic pressure may perturb Starling forces and thereby promote pleural effusion. This mechanism may itself contribute to the excess 30-day mortality observed in the RCP cohort: Schiefenhövel and colleagues demonstrated that postoperative pleural effusions requiring secondary drainage were associated with increased mortality among cardiac surgical patients, a relationship that may also account for the longer, though statistically non-significant, length of stay in the RCP cohort, with implications for resource utilization and overall cost [27].

The majority of thoracic aortic studies focus on elective repairs, whose populations differ substantially from the emergent ATAAD population with respect to age, sex, race, and comorbidities including hypertension, heart failure, obesity, and coagulopathy [28]. Among the few analyses to investigate emergent rather than elective repair, O'Hara and colleagues examined ATAAD patients within the Society of Thoracic Surgeons Adult Cardiac Surgery database and reported that cerebral perfusion significantly reduced composite stroke and mortality relative to circulatory arrest without perfusion, while finding no significant difference in this composite between the RCP and ACP cohorts [3]. In the present analysis, both cohorts underwent the same emergent ATAAD repair with comparable cardiopulmonary bypass, circulatory arrest, and cross-clamp times, with only a modest difference in nadir hypothermic temperature (median = 25°C in both groups; p = 0.027) that was accounted for in multivariable adjustment. Despite this concordance in pre- and intraoperative characteristics, retrograde perfusion was associated with increased risk of 30-day mortality, pericardial/pleural effusion, and cerebrovascular accident, suggesting that the direction of cerebral blood flow and the requirements of each technique may substantially impact clinical outcomes. Because perfusion strategy was not randomized, these findings indicate an association rather than a causal relationship, and the possibility of residual confounding cannot be excluded.

Overall, these findings favor ACP over RCP in the setting of ATAAD repair. This study has several limitations. First, it is a retrospective, single-center analysis and is therefore subject to selection bias and confounding by indication; in particular, the assignment of antegrade versus retrograde perfusion was determined by surgeon discretion rather than by standardized, prespecified criteria, and the retrograde cohort was significantly older than the antegrade cohort. Second, the sample size was modest, and the retrograde cohort in particular was small (n = 20). Third, follow-up was limited to the early (30-day/in-hospital) postoperative period; long-term survival and neurocognitive outcomes were not assessed and represent an important direction for future investigation. Finally, variation in operative technique and the non-randomized nature of strategy selection may limit generalizability to other centers. These limitations underscore the need for larger, prospective, multicenter studies with standardized perfusion protocols to confirm our findings. Future investigation should clarify how non-physiologic retrograde flow influences tissue perfusion, thrombus formation, and clearance, and how cannulation site and perfusion pressure contribute to postoperative outcomes. Given the widespread utilization of cerebral perfusion adjuncts during hypothermic circulatory arrest and increased use of ACP, describing the differences between ACP and RCP remains essential to optimizing outcomes following ATAAD repair.

## Conclusions

In this single-center retrospective analysis of ATAAD repair, the use of RCP was associated with higher rates of 30-day mortality, postoperative pericardial and pleural effusions, and cerebrovascular accident compared with ACP, despite broadly comparable perioperative characteristics between the two cohorts. These findings suggest that important physiologic differences between cerebral perfusion strategies may contribute to meaningful variation in postoperative outcomes. Given the ongoing debate regarding the optimal method of cerebral protection during circulatory arrest, further investigation is warranted. Prospective, multicenter studies with strict controls for confounding variables are needed to better define the short- and long-term clinical implications of cerebral perfusion strategy and to inform evidence-based decision-making in the surgical management of ATAAD.
